# Influence of *FADS* Polymorphisms on Tracking of Serum Glycerophospholipid Fatty Acid Concentrations and Percentage Composition in Children

**DOI:** 10.1371/journal.pone.0021933

**Published:** 2011-07-27

**Authors:** Claudia Glaser, Peter Rzehak, Hans Demmelmair, Norman Klopp, Joachim Heinrich, Berthold Koletzko, the LISA study group

**Affiliations:** 1 Division of Metabolic and Nutritional Medicine, Dr. von Hauner Children's Hospital, University of Munich Medical Center, Munich, Germany; 2 Institute of Epidemiology, Helmholtz Zentrum München, German Research Center for Environmental Health, Neuherberg, Germany; 3 Institute of Medical Informatics, Biometry and Epidemiology, Ludwig-Maximilians University Munich, Munich, Germany; National Institutes of Health, United States of America

## Abstract

**Background:**

Tracking of fatty acid (FA) contribution to plasma or serum lipids over time was shown in children and adults. However, the potential role of *FADS* gene variants has not been investigated.

**Methods and Principal Findings:**

Serum GP FA composition of 331 children aged 2 and 6 years, participating in an ongoing birth cohort study, was analyzed. Correlation coefficients were estimated to describe FA tracking over 4 years and to assess the influence of *FADS* variants on tracking. We found low to moderate tracking (r = 0.12–0.49) of FA compositions and concentration between 2 and 6 years. Concentration changes of total monounsaturated FA and total saturated FA over time correlated closely (r = 0.79) but percentage values were unrelated (r = −0.02). Tracking for n-6 long chain polyunsaturated fatty acid (LC-PUFA) concentrations was lower in subjects homozygous for the major allele of *FADS* variants and higher in carriers of at least one minor allele, whereas for total n-3 LC-PUFA concentrations and compositions this was vice versa. For individual n-3 PUFA inconsistent results were found.

**Conclusions and Significance:**

Serum GP FA composition shows low to moderate tracking over 4 years with a higher tracking for LC-PUFA metabolites than for their precursor FA. Serum PUFA levels and their tracking seem to be more influenced by lipid and lipoprotein metabolism than by FA specific pathways.

## Introduction

Biomarkers of fatty acid (FA) status are widely used in observational studies. They reflect a combination of dietary intake and metabolism. Associations of FA status with current and future health indicators have been demonstrated [1]–[2]. Epidemiological and clinical studies have revealed associations between FA and cardiovascular diseases, diabetes, and certain types of cancer [3]–[5].

The concentrations of individual FA in plasma and tissues do not evolve independently, but rather mutually influence each other. As individual FA are preferentially partitioned into specific lipid pools [6], the percentage FA composition of specific compartments is widely used to describe FA status. Although analyses of FA status are successfully applied for the evaluation of dietary intake [7], FA status is influenced by both diet and endogenous metabolism. In several studies strong associations were found between variants in the human genes *fatty acid desaturase 1 (FADS1)* and *fatty acid desaturase 2 (FADS2)* and blood levels of polyunsaturated fatty acids (PUFA) [8]–[13]. These associations clearly indicate an influence of endogenous metabolism on the blood levels of essential FA. The importance of endogenous metabolism is even greater for long chain polyunsaturated fatty acids (LC-PUFA) than for the saturated and monounsaturated FA which can be synthesized de novo by human metabolism [14].

FA status and in particular the balance of n-3 and n-6 LC-PUFA have been related to long term health [15]–[16]. LC-PUFA have been demonstrated in all lipid compartments considered so far as indicative of human FA status. Their concentrations are highest in glycerophospholipids (GP), which suggests their suitability for describing sensitively FA status including LC-PUFA status. Due to the differences of LC-PUFA concentrations in different compartments (e.g. plasma lipid fractions), analysis of defined, purified fractions is mandatory in order to avoid misleading results.

Another important aspect when evaluating a biomarker is temporal variability, which depends on the turnover of the compartment. The composition of plasma lipids varies within days or weeks, whereas significant changes of FA composition in adipose tissue are only observed after months [7]. As plasma lipid composition may change within short periods, an investigation of the tracking of plasma lipid FA status over years yields information on longer term changes of dietary habits and life style factors. In adults it has been shown that FA composition of plasma cholesteryl esters and phospholipids showed a high degree of tracking, with coefficients of correlation between FA percentages up to 0.83 [17]–[18]. Similar values were observed in total plasma phospholipids of a small group of Portuguese children (n = 26). However, this observation might be very much influenced by the given cultural and socioeconomic conditions of this particular group of children [19].

We aimed to reevaluate the findings of Guerra et al. [19] in a larger, population-based sample of children. Furthermore, we aimed to investigate the influence of polymorphisms in the *FADS* gene cluster on FA tracking which has not been investigated so far.

According to current knowledge, the carriers of the major alleles of the 5 studied SNPs (rs174545, rs174546, rs174556, rs174561, rs3834458) should have a higher conversion of LA and ALA to their corresponding derivates than carriers of at least one minor allele [8], [11]–[12]. We hypothesized that carriers of the major allele would show a greater stability of LC-PUFA values over time and therefore show a greater degree of LC-PUFA tracking than carriers of at least one minor allele.

## Methods

### Ethics statement

For this study, approval by the respective local Ethics Committees (Bavarian General Medical Council, University of Leipzig, Medical Council of North-Rhine-Westphalia) and written informed consent from the families (parents) of participants were obtained.

### Study design and population

The LISA study (“Influences of Lifestyle related Factors on the Immune System and the Development of Allergies in Childhood”) is an ongoing population-based birth cohort study of unselected newborns. The design of this study has been described elsewhere before [20]–[21]. Between November 1997 and January 1999, 3097 healthy full-term newborns were recruited in Munich (n = 1467), Leipzig (n = 976), Wesel (n = 306), and Bad Honnef (n = 348). Neonates were excluded if at least one of the following exclusion criteria was present: preterm birth (maturity <37 gestational week), low birth weight (<2500 g), congenital malformation, symptomatic neonatal infection, antibiotic medication, hospitalisation or intensive medical care during neonatal period. In addition to lack of consent to participate in this study, newborns from mothers with immune-related diseases such as autoimmune disorders, diabetes, hepatitis B, long-term medication or abuse of drugs and alcohol, and newborns from parents with a nationality other than German and parents who were not born in Germany were excluded.

We collected questionnaire data on family history of atopy, parental education and health problems during pregnancy, smoking of the mother during pregnancy and mothers' exposure to environmental tobacco smoke during pregnancy at home. The cohort was followed up at the ages of 6, 12 and 18 months, and 2, 4, 6 and 10 years. Blood samples were collected at birth (cord blood) and from the children at 2, 6 and 10 years.

**Figure 1:**
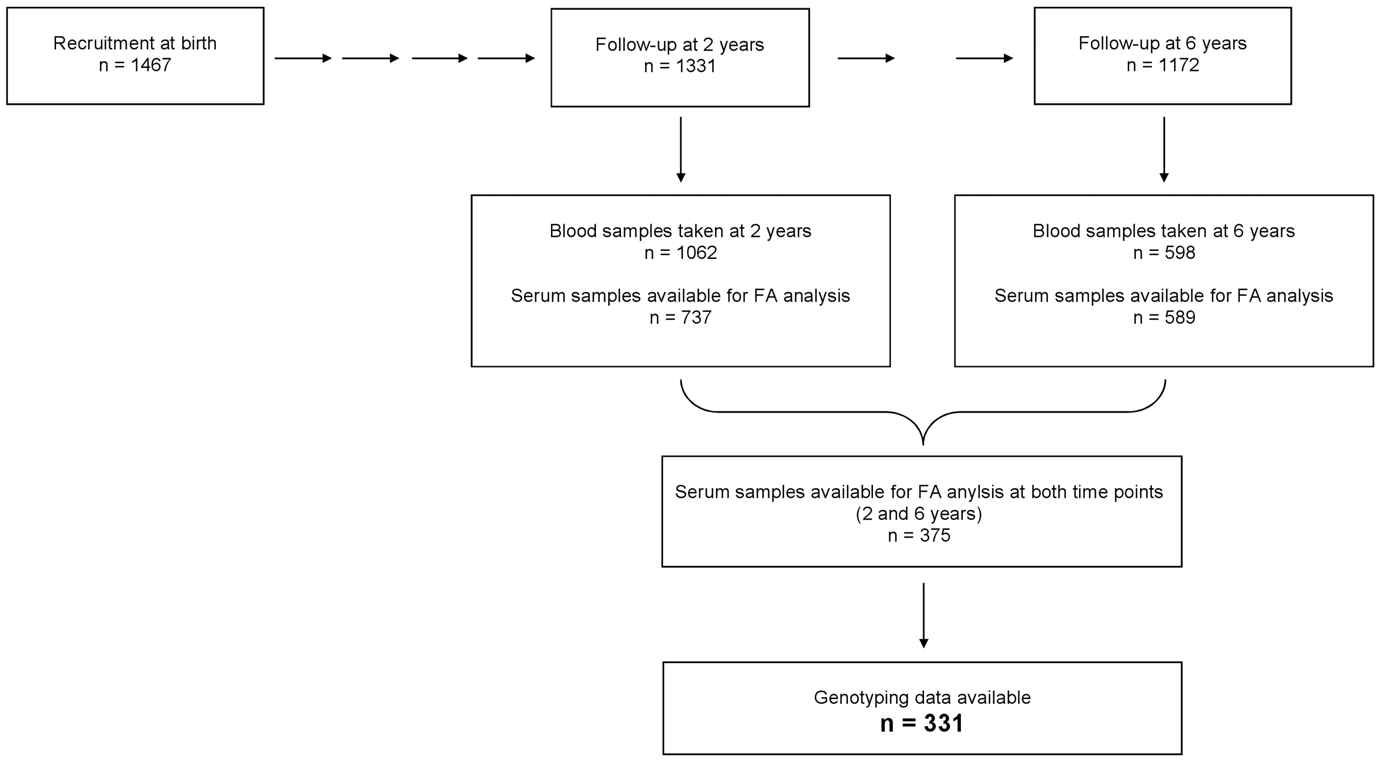
Analyzed serum samples and genetic measurements from the Munich LISA cohort.

For the present analyses, only data from the 2- and 6-year follow-up of study center Munich (n = 1331 and n = 1172, respectively) were included. Serum samples both at the age of 2 and 6 years were available for analysis in 375 children (213 boys, 162 girls). Both genotyping data and fatty acid data were available for 331 children. **Figure 1** shows the origin of the samples.

### Fatty acid analysis

Venous blood samples were collected in serum separator tubes and centrifuged. Serum was frozen in plastic vials and stored at −80°C until analysis. Glycerophospholipid FA were analyzed as described earlier [22]. Briefly, 100 µl of internal standard (1,2-dipentadecanoyl-sn-glycero-3-phosphocholine dissolved in methanol) and 0.6 ml methanol were added to 100 µl of serum, and samples were shaken for 30 s. After centrifugation the supernatant was transferred into another glass tube. Twenty-five µl of sodium methoxide solution were added and tubes were shaken for synthesis of fatty acid methyl esters (FAME). The transesterification was stopped after 3 min by adding 75 µl methanolic HCl. FAME were extracted twice with 300 µl hexane each. Extracts were combined and dried under nitrogen flow at room temperature. The residue was taken up in 50 µl hexane (containing 2 g/l butylated hydroxy toluene) and analyzed by gas chromatography (GC).

We used GC with flame ionization detection for quantifying the FAME. Individual FAME were identified by comparison with authentic standards (GLC-569B, Nu-Check Prep, Inc., Elysian, MN, USA; *cis*-5,8,11-eicosatrienoic acid methyl ester, Sigma-Aldrich, Taufkirchen, Germany; methyl vaccenate (11c), methyl octadecatetraenoate (6c, 9c, 12c, 15c) and methyl brassidate (13tr), Larodan Fine Chemicals AB, Malmö, Sweden). We used the general designations C16:1t, C18:1t, C18:2tt and C22:1t in the results section because we cannot exclude that different isomeric *trans* FA coelute.

As external standard GLC-85 (Nu-Check Prep, Inc., Elysian, MN, USA) was used for determining the response relative to pentadecanoic acid methyl ester (internal standard). For peak integration EZChrom Elite version 3.1.7 (Agilent, Waldbronn, Germany) was used.

### Genotyping

Genomic DNA was extracted from EDTA blood using standard methods and amplified by using REPLI-g UltraFast technology (Qiagen™). Five variants of the *FADS1 FADS2* gene cluster (rs174545, rs174546, rs174556, rs174561, rs3834458) were typed, which have been previously shown to be in strong linkage disequilibrium with each other [8], [11]. SNPs were selected based on previous publications [8], [11]–[12]. Applying the tagger server program (http://www.broadinstitute.org/mpg/tagger/) in combination with HapMap we found that with the 3 SNPs rs174545, rs174546 and rs174556 27 SNPs between basepair positions 61234329 and 61372379 of *FADS1 FADS2* could be tagged. The efficiency was 10.7 fold even though the two further SNPs rs174561 and rs3834458 could not be included as these are not included in the HapMap database. Genotyping of SNPs was realized with the iPLEX (Sequenom, San Diego, CA, USA) method by means of matrix assisted laser desorption ionization-time of flight mass spectrometry method (MALDI-TOF MS, Mass Array; Sequenom) according to the manufacturer's instructions. Standard genotyping quality control included 10% duplicate and negative samples. Genotyping discordance rate was below 0.3%.

### Statistical analysis

For the studied subpopulation allele frequencies and Fisher's exact test of Hardy-Weinberg-Equilibrium were conducted with procedure “proc allele” of the statistical software module SAS/GENETICS of SAS version 9.1.3. Lewontin's D' and pairwise-squared correlations r^2^ were calculated with the software JLIN [23] to examine linkage disequilibrium.

FA data are presented as medians and interquartile ranges (IQR, ranges from the 1st to the 3rd quartile), since FA with very low concentrations showed skewed distributions.

Level of tracking between FA data obtained at the two time points was estimated by Spearman correlation. This is the simplest way for continuous outcome variables to asses tracking between two measurements [24]. Furthermore, Spearman correlation coefficients were calculated for the single SNPs applying an additive model where homozygous minor allele carriers were coded as 2, heterozygous coded as 1, and homozygous major allele carriers coded as 0 ( = reference category). Spearman correlations were performed using the statistical software PASW Statistics, version 18.0.0.

## Results

Baseline characteristics for the total study population and for the studied subpopulation of the LISA Munich cohort are listed in **Table 1**. Weight, height and BMI are comparable between the total and the subpopulation. However, the percentage of boys, fully breastfeeding and high maternal education is somewhat higher and the proportion of maternal smoking during pregnancy is slightly lower in the studied subpopulation.

**Table 1:** Baseline characteristics of the total study population and the studied subpopulation of the LISA Munich cohort.

	Total population			Studied subpopulation		
	n	mean or %	SD	n	mean or %	SD
% boys	1467	52.7		331	55.6	
% girls	1467	47.3		331	44.4	
birth weight (kg)	1466	3.4	0.4	331	3.4	0.4
birth length (cm)	1445	52.1	2.4	326	52.0	2.3
% fully breastfed for at least 4 months	1371	68.7		329	71.4	
% high maternal education	1457	63.9		328	66.5	
% maternal smoking during pregnancy	1458	14.2		328	11.0	

Information regarding position, possible functional region, genotyping frequencies and *P*-values of Fisher's exact test for violation of Hardy-Weinberg-Equilibrium for the five analyzed SNPs of the *FADS1 FADS2* gene cluster are given in **Table 2**. The minimum *P*-value for any of the five analyzed SNPs was 0.69 (rs174556 and rs174561).

**Table 2:** Characteristics of the five analyzed variants in *FADS1 FADS2* gene of the studied subpopulation (n = 331).

dbSNP	Position	Possible functional region (on chromosome 11)	Alleles (major/minor)	Number (%) of subjects with genotype			*P*-value (Fischer's exact test)
	bp		1/2	11	12	22	
rs174545	61325882	*FADS1 UTR-3*	C/G	157 (47.6)	139 (42.1)	34 (10.3)	0.70
rs174546	61326406	*FADS1 UTR-3*	C/T	158 (47.7)	139 (42.0)	34 (10.3)	0.70
rs174556	61337211	*FADS1 intron 2*	C/T	166 (50.5)	138 (41.9)	25 (7.6)	0.69
rs174561	61339284	*FADS1 intron 1*	T/C	166 (50.5)	138 (41.9)	25 (7.6)	0.69
rs3834458	61351497	*FADS2 5′ flanking*	T/Del	154 (47.1)	142 (43.4)	31 (9.5)	0.90

Note: Del indicates deletion; SNP build 131 accessed 27 April 2010, Map to Genom Build 36.3, 11 = homozygous major, 12 = heterozygous, 22 = homozygous minor.

**Figure 2:**
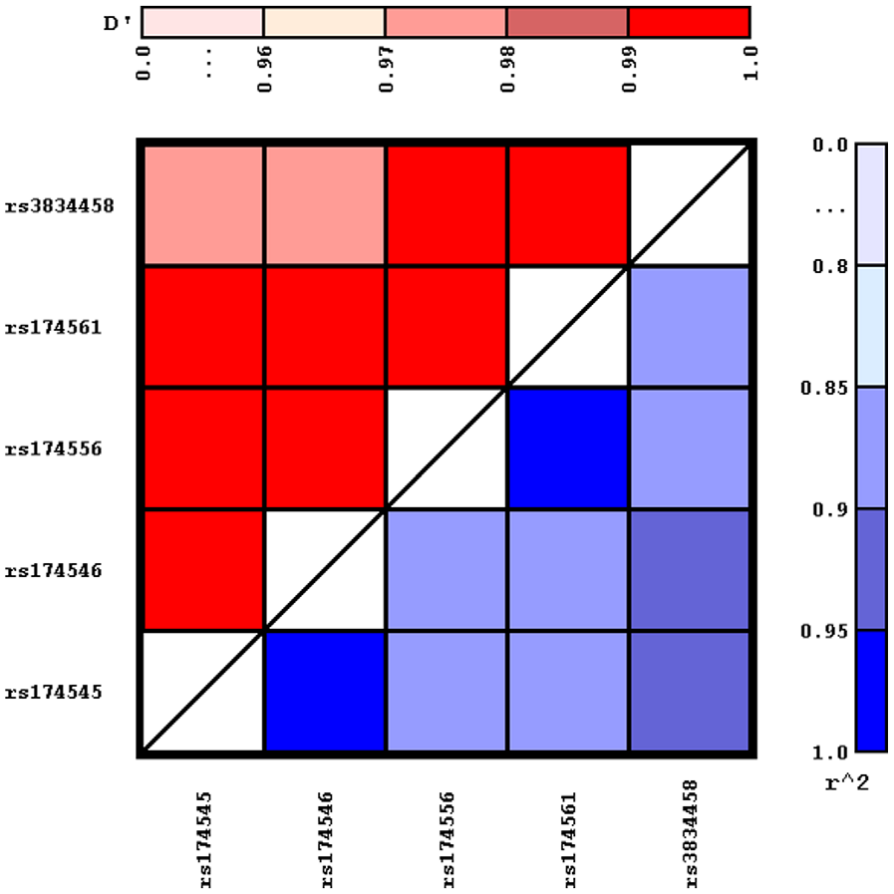
Pair wise linkage disequilibrium measured by Lewontin's D' and r^2^ for the common five single nucleotide polymorphisms (SNP) in the studied subpopulation (n = 331) of the LISA Munich cohort.

Lewontin's D' and pairwise-squared correlations r^2^ for the studied subpopulation are shown in **Figure 2**. Lewontin's D' ranged between 0.97 and 1.0 and the pairwise-squared correlations r^2^ ranged between 0.87 and 1.0, confirming that all five SNPs are in high linkage disequilibrium.

### Fatty acid composition of serum GP

Five FA, namely the saturated FA palmitic (C16:0) and stearic acid (C18:0), the monounsaturated FA oleic acid (C18:1n-9) and the two n-6 PUFA linoleic acid (LA, C18:2n-6) and arachidonic acid (AA, C20:4n-6), showed the highest concentrations in serum GP and accounted for more than 85% of total GP FA (**Table S1**). Up to 45% of all serum GP FA were saturated, ∼40% were polyunsaturated, and ∼15% were monounsaturated. *Trans* FA were found in minor quantities, contributing less than 0.5% to total GP FA. The abundance of n-6 PUFA was ∼7 times higher than that of n-3 PUFA in GP.

Concentrations of total saturated fatty acids (SFA), total monounsaturated fatty acids (MUFA) and PUFA were higher at 6 years compared to 2 years, while the concentration of total *trans* FA did not differ with time. Percentage values did not differ significantly between both time points. However, the PUFA/SFA ratio increased from 0.92 at 2 years to 0.96 at 6 years. N-6 PUFA showed higher values at 6 years compared to 2 years, while n-3 PUFA values remained constant. The n6/n3 ratio increased from 7.5 to 8.2 over time.

**Figure 3:**
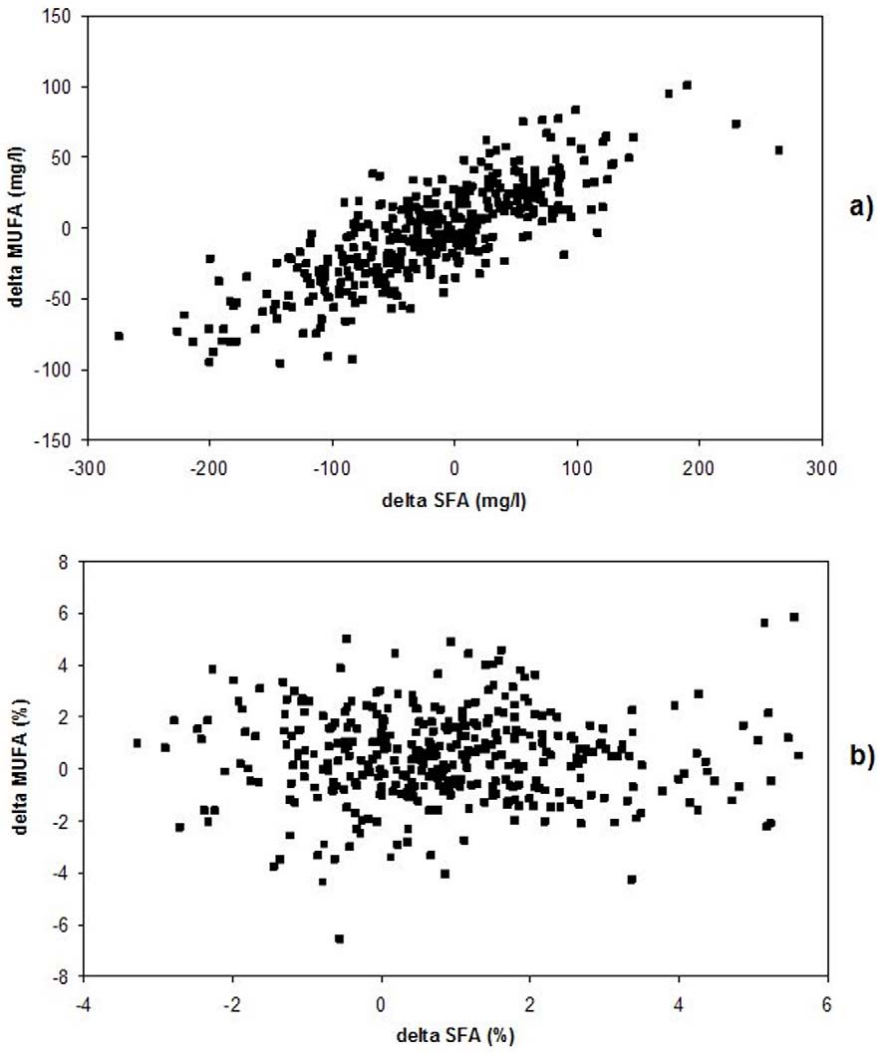
Changes of total monounsaturated fatty acids between 2 and 6 years (delta MUFA, mg/l) show a high correlation (r = 0.79) with changes of total saturated fatty acids (delta SFA, mg/l) (a), while changes of corresponding percentage values (%) are unrelated (b, r = −0.02).

Changes in concentrations of individual FA or groups of FA were positively correlated with each other for most FA (data not shown), e.g. changes of LA concentrations from 2 to 6 years were highly correlated with changes of the concentrations of total SFA (0.71***), total MUFA (0.49***), C18:3n-6 (0.30***), C20:3n-6 (0.25***), C20:4n-6 (0.41***) and C18:3n-3 (0.43***). For percentage values changes in LA were negatively correlated with the changes in the percentage values of total SFA (−0.50***), total MUFA (−0.40***), C20:3n-6 (−0.45***), C20:4n-6 (−0.34***), C20:5n-3 (−0.35***), and C22:6n-3 (−0.37***). Besides the correlation with LA, total SFA and total MUFA percentage values were not correlated with other FA percentage values (data not shown). The changes of total SFA concentrations over time were highly correlated with the changes of total MUFA concentrations (r = 0.79***, **Figure 3a**), whereas no correlation was observed between the changes of total SFA and total MUFA percentages over the four year period (r = −0.02, **Figure 3b**).

### Tracking

For most FA the percentage contributions and concentrations (**Table S1**) at the age of 2 years were significantly correlated to those at 6 years. FA concentrations were stronger correlated between both time points than FA percentage contributions. The highest correlations between both time points were observed for the n-6 LC-PUFA dihomo-gamma-linolenic acid (DGLA, C20:3n-6), AA and docosapentaenoic acid (DPA, C22:5n-6) for both, concentrations and percentage values.

Moderate tracking (0.6>r>0.4) was observed for the percentage values of four FA (C17:0, C20:3n-6, C20:4n-6, and C22:5n-6) and for the concentrations of six FA (C17:0, C18:0, C20:3n-6, C20:4n-6, C22:5n-6, and C22:5n-3), respectively. The majority of all analyzed FA showed low or no tracking (r<0.4).

Concentrations of SFA in serum GP showed a higher degree of tracking than their corresponding percentage values. Interestingly, total SFA percentage values showed no correlation whereas single SFA showed low to moderate tracking. For n-6 PUFA moderate correlations between both time points were observed, with the highest correlations for DGLA, and weaker correlations for their precursor FA LA and gamma-linoleic acid (GLA, C18:3n-6). Tracking of n-3 PUFA was markedly lower than that of n-6 PUFA, with lower tracking rates for the precursor n-3 PUFA compared to their longer chain derivates. Similar findings were observed for n-9 FA. Percentage values of mead acid (20:3n-9) were higher correlated between both time points (r = 0.39) than those of oleic acid (r = 0.22), a precursor of mead acid. *Trans* FA and MUFA had weak correlations.

The ratio of the n-6 PUFA AA/LA showed a moderate degree of tracking (r = 0.41), and the ratio remained practically constant over the 4 year period with 0.42±0.13 at 2 years, and 0.40±0.11 at 6 years, respectively. There was an increasing trend over time for the n-3 PUFA ratios EPA/ALA (2 years: 2.31±1.27; 6 years: 2.50±1.51) and DHA/ALA (2 years: 12.72±8.23; 6 years: 13.58±8.34). Compared to the n-6 PUFA ratio of AA/LA somewhat lower tracking levels were observed for the n-3 PUFA ratios EPA/ALA (r = 0.20) and DHA/ALA (r = 0.20), respectively.

### Influence of FADS polymorphisms on tracking

We found that tracking of n-6 LC-PUFA concentrations (except for C22:4n-6) was higher for homozygous and heterozygous carriers of minor alleles in the five analyzed SNPs than for homozygous carriers of major alleles (**Table S2**). In contrast, tracking of n-6 LC-PUFA compositions showed no clear trend. For total n-3 LC-PUFA concentrations and compositions, minor allele carriers had a lower tracking compared to homozygous major allele carriers but individual n-3 LC-PUFA showed no consistent trend. Tracking of C20:3n-3 and EPA was lower in minor allele carriers than in carriers homozygous for the major allele, whereas results for C22:5n-3 were inconsistent. For DHA tracking was comparable between the 3 groups in all analyzed SNPs.

## Discussion

### Fatty acid status

We found increasing serum GP total FA concentrations over the 4-year follow-up. The children studied had higher serum PUFA concentrations at the age of 6 than at 2 years, whereas SFA and MUFA concentrations showed only a slight increase over time. Hence, the PUFA/SFA ratio increased with age. These observations point towards a change in food habits with a shift to higher proportions of polyunsaturated dietary fat. Our findings are in accordance with observations from Guerra et al. [19] who found an increase of plasma phospholipid PUFA/SFA ratio in 26 Portuguese children from 2 to 5 years of age.

Plasma GP n-6 PUFA concentrations increased during the follow-up, which likely reflects a higher intake of foods providing LA or AA (e.g. vegetable oils, eggs, meat and meat products) at older age [25]. In contrast, plasma concentrations of n-3 PUFA remained almost constant over time. This is presumably attributable to a low consumption of n-3 PUFA in the diets of most German children. The main dietary sources of n-3 LC-PUFA are fish and other seafood. Children who like to eat fish and whose parents pay attention to a regular fish intake are likely to maintain such habits over time, and a relatively high n-3 LC-PUFA level will persist; whereas children who tend not to eat fish retain a low n-3 LC-PUFA level. Our findings suggest that only few children change their food habits with regard to fish intake over time.

The strong positive correlation between the changes in the concentrations of total MUFA and total SFA, points towards a strong influence of lipid metabolism on FA concentrations. This assumption is strengthened by the absence of a correlation between changes in the percentage values of total MUFA and total SFA, as well as previous findings of Moilanen et al. [26] who reported a significant association between serum cholesteryl ester FA composition and various serum lipids.

LA values seem to have a strong influence on the percentage composition of serum GP FA, whereas the impact of SFA and MUFA on FA composition is very low. The correlations between the changes in FA percentages over the 4-year period indicate that the changes in LA mainly affected SFA and MUFA. We found negative correlations of LA with C20:3n-6, C20:4n-6, C20:5n-3 and C22:6n-3, which was in accordance with previous findings [27].

### Tracking

Tracking of FA percentage values in plasma or serum phospholipids and cholesteryl esters has previously been reported in children and adults [17]–[19], [27], but tracking of FA concentrations has not been investigated before. Our results revealed tracking rates of similar magnitude compared to findings of Guerra et al. [19] for total SFA (0.02/−0.02, our results/Guerra et al.) and total MUFA (0.22/0.25), whereas n-6 LC-PUFA (0.35/0.63) and n-3 LC-PUFA (0.23/0.31) showed a somewhat lower tracking in our study. This might be explained by the different study settings. Guerra et al. examined tracking of plasma phospholipid FA in 26 Portuguese children between the age of 2 and 5 years, whereas we examined tracking of FA in serum GP (a subgroup of phospholipids) in a larger group of 331 German children between the ages of 2 and 6 years. Previous publications show that tracking of plasma cholesteryl ester FA is higher than that of plasma phospholipid FA, and that FA tracking might be higher in adults than in children [17]–[18], [28]. However, independent from the studied subjects and the analyzed lipid fraction, our results are consistent with previously published data on FA tracking in that n-6 LC-PUFA showed greater tracking than n-3 LC-PUFA, and that LA and ALA have a lower degree of tracking than their LC-PUFA derivates.

We also determined tracking of FA concentrations, which turned out to be somewhat greater than the tracking of FA percentages, particularly for SFA. This further supports that lipid metabolism might have a significant influence on serum GP FA concentrations. The high tracking of C17:0, which reflects the intake of dairy fats, might indicate a stable dietary milk and dairy product intake. Patterson et al. [29] reported a tracking correlation coefficient of 0.3 for dietary intakes of milk and yoghurt in Swedish children over a period of 6 years.

Our hypothesis that carriers of the major allele show higher tracking than carriers of at least one minor allele could be confirmed only for serum GP total n-3 LC-PUFA levels. For individual n-3 PUFA this trend is not consistent. In contrast to our expectation, tracking of total n-6 LC-PUFA percentage and concentration values was lower in subjects homozygous for the major allele than in subjects carrying one minor allele. However, tracking values were comparable between carriers homozygous for the major allele and carriers homozygous for the minor allele, except for SNP rs3834458, here tracking values of subjects homozygous for the minor allele were comparable with theses of subjects carrying one minor and one major allele.

We assume that the major portion of serum GP n-6 LC-PUFA levels are contributed by endogenous synthesis via the desaturase/elongase pathway, which was shown to be largely influenced by genetic variants in the *FADS1 FADS2* gene cluster [11]. Linoleic acid, the n-6 LC-PUFA precursor, is the most abundant PUFA in diet and serum lipids. Serum GP LA values are largely determined by dietary intake. In our study we found an increase of LA levels over time and only slight tracking for LA values. Thus, subjects with higher LA conversion rates have a higher variability in n-6 LC-PUFA levels depending on available LA and therefore tend to show a lower tracking. The hypothesized association of higher tracking with higher conversion intensity can only be observed for very low precursor levels, as is the case for n-3 PUFA.

In conclusion, the present study documents low to moderate tracking of serum GP FA composition over a period of 4-years. The highest tracking was observed for n-6 LC-PUFA. Furthermore, LC-PUFA metabolites have a higher tracking than their precursor FA. We found that *FADS1 FADS2* gene variants modulate tracking of serum GP PUFA levels. However, results are inconsistent and the influence of general lipid and lipoprotein metabolism seems to be more pronounced than the influence of FA specific pathways. As a consequence FA concentrations show a higher degree of tracking than FA percentage values.

## Supporting Information

Table S1 **Tracking of serum glycerophospholipid fatty acids between both time points of the studied subpopulation (n = 331) estimated by Spearman correlations.**
(DOCX)

Table S2 **Influence of FADS1 FADS2 gene variants on tracking of serum glycerophospholipid PUFA levels between both time points of the studied subpopulation (n = 331) estimated by Spearman correlations.**
(DOCX)
