# Supplementary material for: Influence of FADS Polymorphisms on Tracking of Serum Glycerophospholipid Fatty Acid Concentrations and Percentage Composition in Children
Source: PLoS One. 2011 Jul 27;6(7):e21933. doi: 10.1371/journal.pone.0021933 (PMC3144869; doi:10.1371/journal.pone.0021933)
Supplement: Table S1 — Tracking of serum glycerophospholipid fatty acids between both time points of the studied subpopulation (n = 331) estimated by Spearman correlations. (DOCX) [file pone.0021933.s001.docx]

|  |  |  |  |  |  |  |  |  |  |
| --- | --- | --- | --- | --- | --- | --- | --- | --- | --- |
| Fatty acid |  | Composition (%) | |  |  |  | Concentration (mg/l) | |  |
|  |  |  | Spearman | |  |  |  | Spearman | |
|  | 2 years | 6 years | R | (Significance) |  | 2 years | 6 years | R | (Significance) |
|  |  |  |  |  |  |  |  |  |  |
|  |  |  |  |  |  |  |  |  |  |
| Saturated FA |  |  |  |  |  |  |  |  |  |
| C14:0 | 0.52 (0.33) | 0.48 (0.25) | 0.15 | (0.006) |  | 5.9 (3.8) | 5.7 (3.5) | 0.17 | (0.002) |
| C16:0 | 28.56 (1.59) | 27.47 (1.42) | 0.28 | (< 0.001) |  | 326.1 (58.3) | 331.1 (67.0) | 0.40 | (< 0.001) |
| C17:0 | 0.42 (0.09) | 0.40 (0.07) | 0.42 | (< 0.001) |  | 4.8 (1.3) | 4.7 (1.3) | 0.45 | (< 0.001) |
| C18:0 | 14.68 (1.36) | 15.07 (1.14) | 0.33 | (< 0.001) |  | 165.3 (36.9) | 180.3 (43.1) | 0.47 | (< 0.001) |
| Sum SFA | 44.13 (1.45) | 43.43 (1.33) | 0.02 | (0.763) |  | 506.6 (91.8) | 517.2 (113.4) | 0.42 | (< 0.001) |
| *Trans* FA |  |  |  |  |  |  |  |  |  |
| C16:1t | 0.03 (0.02) | 0.03 (0.01) | 0.26 | (< 0.001) |  | 0.4 (0.1) | 0.3 (0.1) | 0.29 | (< 0.001) |
| C18:1t | 0.17 (0.08) | 0.17 (0.09) | 0.18 | (< 0.001) |  | 1.9 (1.0) | 2.1 (1.0) | 0.19 | (< 0.001) |
| C18:2tt | 0.09 (0.04) | 0.08 (0.04) | 0.28 | (< 0.001) |  | 1.1 (0.5) | 1.0 (0.5) | 0.33 | (< 0.001) |
| C22:1t | 0.10 (0.05) | 0.10 (0.05) | 0.20 | (< 0.001) |  | 1.1 (0.8) | 1.2 (0.7) | 0.2 | (< 0.001) |
| Sum *trans*FA | 0.42 (0.16) | 0.41 (0.14) | 0.22 | (< 0.001) |  | 4.8 (2.1) | 4.9 (1.8) | 0.26 | (< 0.001) |
| Cis monounsaturated FA |  |  |  |  |  |  |  |  |  |
| C15:1n-5 | 0.07 (0.03) | 0.07 (0.03) | 0.28 | (< 0.001) |  | 0.8 (0.4) | 0.8 (0.2) | 0.32 | (< 0.001) |
| C16:1n-7 | 0.58 (0.23) | 0.55 (0.21) | 0.31 | (< 0.001) |  | 6.3 (3.3) | 6.6 (2.8) | 0.31 | (< 0.001) |
| C18:1n-7 | 1.29 (0.21) | 1.26 (0.19) | 0.33 | (< 0.001) |  | 14.4 (3.4) | 15.2 (3.3) | 0.36 | (< 0.001) |
| C18:1n-9 | 12.09 (1.77) | 11.84 (1.64) | 0.22 | (< 0.001) |  | 136.6 (37.9) | 139.7 (36.8) | 0.25 | (< 0.001) |
| C20:1n-9 | 0.13 (0.04) | 0.14 (0.03) | 0.12 | (0.031) |  | 1.5 (0.6) | 1.7 (0.6) | 0.23 | (< 0.001) |
| Sum MUFA | 14.17 (1.76) | 13.88 (1.79) | 0.22 | (0.603) |  | 160.9 (42.2) | 165.0 (41.7) | 0.27 | (< 0.001) |
| n-9 PUFA |  |  |  |  |  |  |  |  |  |
| C20:3n-9 | 0.23 (0.13) | 0.21 (0.08) | 0.39 | (< 0.001) |  | 2.5 (1.7) | 2.5 (1.1) | 0.34 | (< 0.001) |
| n-6 PUFA |  |  |  |  |  |  |  |  |  |
| C18:2n-6 | 22.22 (3.59) | 23.60 (3.38) | 0.27 | (< 0.001) |  | 252.7 (63.3) | 282.8 (78.7) | 0.32 | (< 0.001) |
| C18:3n-6 | 0.09 (0.05) | 0.10 (0.05) | 0.29 | (< 0.001) |  | 1.0 (0.7) | 1.2 (0.7) | 0.25 | (< 0.001) |
| C20:2n-6 | 0.30 (0.08) | 0.30 (0.06) | 0.22 | (< 0.001) |  | 3.4 (1.0) | 3.5 (1.0) | 0.31 | (< 0.001) |
| C20:3n-6 | 2.89 (0.79) | 2.99 (0.70) | 0.49 | (< 0.001) |  | 32.4 (9.9) | 36.2 (10.6) | 0.47 | (< 0.001) |
| C20:4n-6 | 9.35 (1.86) | 9.54 (1.60) | 0.43 | (< 0.001) |  | 104.4 (25.3) | 114.8 (24.9) | 0.45 | (< 0.001) |
| C22:4n-6 | 0.40 (0.11) | 0.37 (0.11) | 0.31 | (< 0.001) |  | 4.5 (1.4) | 4.5 (1.2) | 0.40 | (< 0.001) |
| C22:5n-6 | 0.44 (0.16) | 0.40 (0.15) | 0.45 | (< 0.001) |  | 5.0 (2.0) | 4.7 (1.8) | 0.48 | (< 0.001) |
| Sum n-6 LC-PUFA | 13.43 (2.12) | 13.59 (2.05) | 0.35 | (< 0.001) |  | 150.2 (31.2) | 162.7 (32.9) | 0.40 | (< 0.001) |
| n-3 PUFA |  |  |  |  |  |  |  |  |  |
| C18:3n-3 | 0.22 (0.10) | 0.21 (0.10) | 0.19 | (< 0.001) |  | 2.6 (1.3) | 2.6 (1.5) | 0.21 | (< 0.001) |
| C20:3n-3 | 0.07 (0.02) | 0.06 (0.01) | 0.13 | (0.019) |  | 0.8 (0.3) | 0.8 (0.2) | 0.27 | (< 0.001) |
| C20:5n-3 | 0.50 (0.29) | 0.53 (0.23) | 0.26 | (< 0.001) |  | 5.8 (3.3) | 6.4 (3.5) | 0.26 | (< 0.001) |
| C22:5n-3 | 0.96 (0.24) | 0.87 (0.23) | 0.32 | (< 0.001) |  | 11.2 (3.4) | 10.6 (3.1) | 0.44 | (< 0.001) |
| C22:6n-3 | 2.97 (0.83 | 2.84 (0.88) | 0.27 | (< 0.001) |  | 33.8 (10.4) | 34.8 (11.4) | 0.37 | (< 0.001) |
| Sum n-3 LC-PUFA | 4.53 (0.89) | 4.34 (1.14) | 0.23 | (< 0.001) |  | 51.0 (13.9) | 53.1 (14.4) | 0.38 | (< 0.001) |
| Total FA |  |  |  |  |  | 1138.4 (217.9) | 1197.6 (244.9) | 0.40 | (< 0.001) |
|  |  |  |  |  |  |  |  |  |  |

Note: FA compositions (%, wt./wt.) and concentrations (mg/l) at 2 and 6 years are presented as median (interquartile range).
