# Supplementary material for: Influence of FADS Polymorphisms on Tracking of Serum Glycerophospholipid Fatty Acid Concentrations and Percentage Composition in Children
Source: PLoS One. 2011 Jul 27;6(7):e21933. doi: 10.1371/journal.pone.0021933 (PMC3144869; doi:10.1371/journal.pone.0021933)
Supplement: Table S2 — Influence of FADS1 FADS2 gene variants on tracking of serum glycerophospholipid PUFA levels between both time points of the studied subpopulation (n = 331) estimated by Spearman correlations. (DOCX) [file pone.0021933.s002.docx]

|  | |  |  |  |  |  |  |  |  |  |  |  |  |  |  |  |
| --- | --- | --- | --- | --- | --- | --- | --- | --- | --- | --- | --- | --- | --- | --- | --- | --- |
|  | | n-6 PUFA | | | | | | | |  | n-3 PUFA | | | | | |
|  | | C18:2n-6 | C18:3n-6 | C20:2n-6 | C20:3n-6 | C20:4n-6 | C22:4n-6 | C22:5n-6 | Sum n-6 |  | C18:3n-3 | C20:3n-3 | C20:5n-3 | C22:5n-3 | C22:6n-3 | Sum n-3 |
|  | |  |  |  |  |  |  |  | LC-PUFA |  |  |  |  |  |  | LC-PUFA |
|  | |  |  |  |  |  |  |  |  |  |  |  |  |  |  |  |
| rs174545 | |  |  |  |  |  |  |  |  |  |  |  |  |  |  |  |
|  | |  |  |  |  |  |  |  |  |  |  |  |  |  |  |  |
| *Compositions* | |  |  |  |  |  |  |  |  |  |  |  |  |  |  |  |
| 11 | R | 0.24 | 0.27 | 0.17 | 0.48 | 0.27 | 0.34 | 0.45 | 0.27 |  | 0.07 | 0.21 | 0.23 | 0.30 | 0.27 | 0.29 |
| n = 157 | (Significance) | (0.003) | (0.001) | (0.034) | (<0.001) | (0.001) | (<0.001) | (<0.001) | (0.001) |  | (0.405) | (0.010) | (0.004) | (<0.001) | (0.001) | (<0.001) |
| 12 | R | 0.28 | 0.26 | 0.16 | 0.47 | 0.40 | 0.30 | 0.43 | 0.38 |  | 0.35 | 0.06 | 0.19 | 0.23 | 0.26 | 0.18 |
| n = 139 | (Significance) | (0.001) | (0.002) | (0.066) | (<0.001) | (<0.001) | (<0.001) | (<0.001) | (<0.001) |  | (<0.001) | (0.516) | (0.029) | (0.006) | (0.002) | (0.039) |
| 22 | R | 0.16 | 0.32 | 0.33 | 0.34 | 0.40 | 0.03 | 0.34 | 0.27 |  | 0.001 | 0.08 | 0.18 | 0.51 | 0.27 | 0.16 |
| n = 34 | (Significance) | (0.354) | (0.061) | (0.059) | (0.053) | (0.020) | (0.864) | (0.050) | (0.125) |  | (0.994) | (0.647) | (0.319) | (0.001) | (0.128) | (0.359) |
|  | |  |  |  |  |  |  |  |  |  |  |  |  |  |  |  |
| *Concentrations* | |  |  |  |  |  |  |  |  |  |  |  |  |  |  |  |
| 11 | R | 0.31 | 0.24 | 0.20 | 0.42 | 0.38 | 0.39 | 0.48 | 0.35 |  | 0.13 | 0.30 | 0.27 | 0.46 | 0.38 | 0.45 |
| n = 157 | (Significance) | (<0.001) | (0.002) | (0.010) | (<0.001) | (<0.001) | (<0.001) | (<0.001) | (<0.001) |  | (0.097) | (<0.001) | (0.001) | (<0.001) | (<0.001) | (<0.001) |
| 12 | R | 0.33 | 0.20 | 0.33 | 0.45 | 0.44 | 0.42 | 0.46 | 0.43 |  | 0.33 | 0.23 | 0.17 | 0.34 | 0.38 | 0.34 |
| n = 139 | (Significance) | (<0.001) | (0.016) | (<0.001) | (<0.001) | (<0.001) | (<0.001) | (<0.001) | (<0.001) |  | (<0.001) | (0.006) | (0.043) | (<0.001) | (<0.001) | (<0.001) |
| 22 | R | 0.27 | 0.23 | 0.55 | 0.47 | 0.41 | 0.26 | 0.54 | 0.38 |  | -0.09 | 0.18 | 0.17 | 0.51 | 0.34 | 0.22 |
| n = 34 | (Significance) | (0.120) | (0.190) | (0.001) | (0.005) | (0.016) | (0.138) | (0.001) | (0.027) |  | (0.601) | (0.314) | (0.337) | (0.002) | (0.048) | (0.213) |
|  | |  |  |  |  |  |  |  |  |  |  |  |  |  |  |  |
| rs174546 | |  |  |  |  |  |  |  |  |  |  |  |  |  |  |  |
|  | |  |  |  |  |  |  |  |  |  |  |  |  |  |  |  |
| *Compositions* | |  |  |  |  |  |  |  |  |  |  |  |  |  |  |  |
| 11 | R | 0.24 | 0.27 | 0.17 | 0.48 | 0.27 | 0.35 | 0.46 | 0.27 |  | 0.07 | 0.20 | 0.23 | 0.29 | 0.27 | 0.28 |
| n = 158 | (Significance) | (0.003) | (0.001) | (0.036) | (<0.001) | (0.001) | (<0.001) | (<0.001) | (0.001) |  | (0.373) | (0.012) | (0.003 | (<0.001) | (0.001) | (<0.001) |
| 12 | R | 0.28 | 0.26 | 0.16 | 0.47 | 0.40 | 0.30 | 0.43 | 0.38 |  | 0.35 | 0.06 | 0.19 | 0.23 | 0.26 | 0.18 |
| n = 139 | (Significance) | (0.001) | (0.002) | (0.066) | (<0.001) | (<0.001) | (<0.001) | (<0.001) | (<0.001) |  | (<0.001) | (0.516) | (0.029) | (0.006) | (0.002) | (0.039) |
| 22 | R | 0.16 | 0.32 | 0.33 | 0.34 | 0.40 | 0.03 | 0.34 | 0.27 |  | 0.001 | 0.08 | 0.18 | 0.51 | 0.27 | 0.16 |
| n = 34 | (Significance) | (0.354) | (0.061) | (0.059) | (0.053) | (0.020) | (0.864) | (0.050) | (0.125) |  | (0.994) | (0.647) | (0.319) | (0.002) | (0.128) | (0.359) |
|  | |  |  |  |  |  |  |  |  |  |  |  |  |  |  |  |
| *Concentrations* | |  |  |  |  |  |  |  |  |  |  |  |  |  |  |  |
| 11 | R | 0.31 | 0.24 | 0.20 | 0.43 | 0.39 | 0.39 | 0.49 | 0.35 |  | 0.14 | 0.31 | 0.27 | 0.46 | 0.38 | 0.45 |
| n = 158 | (Significance) | (<0.001) | (0.002) | (0.011) | (<0.001) | (<0.001) | (<0.001) | (<0.001) | (<0.001) |  | (0.082) | (<0.001) | (0.001) | (<0.001) | (<0.001) | (<0.001) |
| 12 | R | 0.33 | 0.20 | 0.33 | 0.45 | 0.44 | 0.42 | 0.46 | 0.43 |  | 0.33 | 0.23 | 0.17 | 0.34 | 0.38 | 0.34 |
| n = 139 | (Significance) | (<0.001) | (0.016) | (<0.001) | (<0.001) | (<0.001) | (<0.001) | (<0.001) | (<0.001) |  | (<0.001) | (0.006) | (0.043) | (<0.001) | (<0.001) | (<0.001) |
| 22 | R | 0.27 | 0.23 | 0.55 | 0.47 | 0.41 | 0.26 | 0.54 | 0.38 |  | -0.09 | 0.18 | 0.17 | 0.51 | 0.34 | 0.22 |
| n = 34 | (Significance) | (0.120) | (0.190) | (0.001) | (0.005) | (0.016) | (0.138) | (0.001) | (0.027) |  | (0.601) | (0.314) | (0.337) | (0.002) | (0.048) | (0.213) |
|  | |  |  |  |  |  |  |  |  |  |  |  |  |  |  |  |
| rs174556 | |  |  |  |  |  |  |  |  |  |  |  |  |  |  |  |
|  | |  |  |  |  |  |  |  |  |  |  |  |  |  |  |  |
| *Compositions* | |  |  |  |  |  |  |  |  |  |  |  |  |  |  |  |
| 11 | R | 0.22 | 0.26 | 0.15 | 0.45 | 0.29 | 0.35 | 0.44 | 0.28 |  | 0.06 | 0.15 | 0.18 | 0.28 | 0.26 | 0.26 |
| n = 166 | (Significance) | (0.004) | (0.001) | (0.050) | (<0.001) | (<0.001) | (<0.001) | (<0.001) | (<0.001) |  | (0.463) | (0.049) | (0.021) | (<0.001) | (0.001) | (0.001) |
| 12 | R | 0.28 | 0.28 | 0.19 | 0.49 | 0.38 | 0.29 | 0.43 | 0.37 |  | 0.37 | 0.11 | 0.26 | 0.27 | 0.27 | 0.16 |
| n = 138 | (Significance) | (0.001) | (0.001) | (0.026) | (<0.001) | (<0.001) | (<0.001) | (<0.001) | (<0.001) |  | (<0.001) | (0.192) | (0.002) | (0.001) | (0.002) | (0.055) |
| 22 | R | 0.32 | 0.38 | 0.41 | 0.27 | 0.40 | 0.08 | 0.46 | 0.29 |  | 0.03 | 0.11 | 0.09 | 0.51 | 0.28 | 0.19 |
| n = 25 | (Significance) | (0.116) | (0.064) | (0.042) | (0.186) | (0.051) | (0.692) | (0.021) | (0.160) |  | (0.882) | (0.602) | (0.669) | (0.009) | (0.168) | (0.371) |
|  | |  |  |  |  |  |  |  |  |  |  |  |  |  |  |  |
| *Concentrations* | |  |  |  |  |  |  |  |  |  |  |  |  |  |  |  |
| 11 | R | 0.30 | 0.24 | 0.22 | 0.44 | 0.41 | 0.41 | 0.49 | 0.37 |  | 0.12 | 0.30 | 0.21 | 0.43 | 0.39 | 0.44 |
| n = 166 | (Significance) | (<0.001) | (0.002) | (0.005) | (<0.001) | (<0.001) | (<0.001) | (<0.001) | (<0.001) |  | (0.126) | (<0.001) | (0.006) | (<0.001) | (<0.001) | (<0.001) |
| 12 | R | 0.34 | 0.21 | 0.33 | 0.44 | 0.42 | 0.40 | 0.45 | 0.41 |  | 0.35 | 0.25 | 0.25 | 0.39 | 0.37 | 0.35 |
| n = 138 | (Significance) | (<0.001) | (0.012) | (<0.001) | (<0.001) | (<0.001) | (<0.001) | (<0.001) | (<0.001) |  | (<0.001) | (0.003) | (0.004) | (<0.001) | (<0.001) | (<0.001) |
| 22 | R | 0.20 | 0.35 | 0.60 | 0.46 | 0.42 | 0.28 | 0.63 | 0.38 |  | -0.05 | 0.18 | 0.07 | 0.48 | 0.38 | 0.25 |
| n = 25 | (Significance) | (0.330) | (0.089) | (0.001) | (0.020) | (0.036) | (0.181) | (0.001) | (0.062) |  | (0.822) | (0.380) | (0.729) | (0.016) | (0.058) | (0.235) |
|  | |  |  |  |  |  |  |  |  |  |  |  |  |  |  |  |
| rs174561 | |  |  |  |  |  |  |  |  |  |  |  |  |  |  |  |
|  | |  |  |  |  |  |  |  |  |  |  |  |  |  |  |  |
| *Compositions* | |  |  |  |  |  |  |  |  |  |  |  |  |  |  |  |
| 11 | R | 0.22 | 0.26 | 0.15 | 0.45 | 0.29 | 0.35 | 0.44 | 0.28 |  | 0.06 | 0.15 | 0.18 | 0.28 | 0.26 | 0.26 |
| n = 166 | (Significance) | (0.004) | (0.001) | (0.050) | (<0.001) | (<0.001) | (<0.001) | (<0.001) | (<0.001) |  | (0.463) | (0.049) | (0.021) | (<0.001) | (0.001) | (0.001) |
| 12 | R | 0.28 | 0.28 | 0.19 | 0.49 | 0.38 | 0.29 | 0.43 | 0.37 |  | 0.37 | 0.11 | 0.26 | 0.27 | 0.27 | 0.16 |
| n = 138 | (Significance) | (0.001) | (0.001) | (0.026) | (<0.001) | (<0.001) | (<0.001) | (<0.001) | (<0.001) |  | (<0.001) | (0.192) | (0.002) | (0.001) | (0.002) | (0.055) |
| 22 | R | 0.32 | 0.38 | 0.41 | 0.27 | 0.40 | 0.08 | 0.46 | 0.29 |  | 0.03 | 0.11 | 0.09 | 0.51 | 0.28 | 0.19 |
| n = 25 | (Significance) | (0.116) | (0.064) | (0.042) | (0.186) | (0.051) | (0.692) | (0.021) | (0.160) |  | (0.882) | (0.602) | (0.669) | (0.009) | (0.168) | (0.371) |
|  | |  |  |  |  |  |  |  |  |  |  |  |  |  |  |  |
| *Concentrations* | |  |  |  |  |  |  |  |  |  |  |  |  |  |  |  |
| 11 | R | 0.30 | 0.24 | 0.22 | 0.44 | 0.41 | 0.41 | 0.49 | 0.37 |  | 0.12 | 0.30 | 0.21 | 0.43 | 0.39 | 0.44 |
| n = 166 | (Significance) | (<0.001) | (0.002) | (0.005) | (<0.001) | (<0.001) | (<0.001) | (<0.001) | (<0.001) |  | (0.126) | (<0.001) | (0.006) | (<0.001) | (<0.001) | (<0.001) |
| 12 | R | 0.34 | 0.21 | 0.33 | 0.44 | 0.42 | 0.40 | 0.45 | 0.41 |  | 0.35 | 0.25 | 0.25 | 0.39 | 0.37 | 0.35 |
| n = 138 | (Significance) | (<0.001) | (0.012) | (<0.001) | (<0.001) | (<0.001) | (<0.001) | (<0.001) | (<0.001) |  | (<0.001) | (0.003) | (0.004) | (<0.001) | (<0.001) | (<0.001) |
| 22 | R | 0.20 | 0.35 | 0.60 | 0.46 | 0.42 | 0.28 | 0.63 | 0.38 |  | -0.05 | 0.18 | 0.07 | 0.48 | 0.38 | 0.25 |
| n = 25 | (Significance) | (0.330) | (0.089) | (0.001) | (0.020) | (0.036) | (0.181) | (0.001) | (0.062) |  | (0.822) | (0.380) | (0.729) | (0.016) | (0.058) | (0.235) |
|  | |  |  |  |  |  |  |  |  |  |  |  |  |  |  |  |
| rs3834458 | |  |  |  |  |  |  |  |  |  |  |  |  |  |  |  |
|  | |  |  |  |  |  |  |  |  |  |  |  |  |  |  |  |
| *Compositions* | |  |  |  |  |  |  |  |  |  |  |  |  |  |  |  |
| 11 | R | 0.25 | 0.29 | 016 | 0.46 | 0.27 | 0.36 | 0.45 | 0.27 |  | 0.06 | 0.19 | 0.25 | 0.29 | 0.27 | 0.28 |
| n = 154 | (Significance) | (0.002) | (<0.001) | (0.045) | (<0.001) | (0.001) | (<0.001) | (<0.001) | (0.001) |  | (0.478) | (0.016) | (0.001 | (<0.001) | (0.001) | (<0.001) |
| 12 | R | 0.26 | 0.25 | 0.16 | 0.46 | 0.41 | 0.33 | 0.43 | 0.38 |  | 0.33 | 0.04 | 0.17 | 0.23 | 0.26 | 0.16 |
| n = 142 | (Significance) | (0.002) | (0.003) | (0.054) | (<0.001) | (<0.001) | (<0.001) | (<0.001) | (<0.001) |  | (<0.001) | (0.637) | (0.047) | (0.005) | (0.002 | (0.062) |
| 22 | R | 0.28 | 0.38 | 0.31 | 0.37 | 0.44 | 0.03 | 0.40 | 0.34 |  | 0.10 | 0.19 | 0.18 | 0.51 | 0.26 | 0.12 |
| n = 31 | (Significance) | (0.125) | (0.037) | (0.086) | (0.042) | (0.014) | (0.883) | (0.026) | (0.065) |  | (0.593) | (0.308) | (0.328) | (0.003) | (0.157) | (0.523) |
|  | |  |  |  |  |  |  |  |  |  |  |  |  |  |  |  |
| *Concentrations* | |  |  |  |  |  |  |  |  |  |  |  |  |  |  |  |
| 11 | R | 0.29 | 0.26 | 0.20 | 0.41 | 0.38 | 0.39 | 0.48 | 0.35 |  | 0.13 | 0.30 | 0.27 | 0.45 | 0.37 | 0.43 |
| n = 154 | (Significance) | (<0.001) | (0.001) | (0.013) | (<0.001) | (<0.001) | (<0.001) | (<0.001) | (<0.001) |  | (0.107) | (<0.001) | (0.001) | (<0.001) | (<0.001) | (<0.001) |
| 12 | R | 0.34 | 0.20 | 0.34 | 0.45 | 0.45 | 0.43 | 0.46 | 0.43 |  | 0.32 | 0.22 | 0.17 | 0.35 | 0.39 | 0.34 |
| n = 142 | (Significance) | (<0.001) | (0.019) | (<0.001) | (<0.001) | (<0.001) | (<0.001) | (<0.001) | (<0.001) |  | (<0.001) | (0.007) | (0.041) | (<0.001) | (<0.001) | (<0.001) |
| 22 | R | 0.23 | 0.31 | 0.53 | 0.51 | 0.47 | 0.34 | 0.61 | 0.45 |  | -0.02 | 0.22 | 0.15 | 0.49 | 0.36 | 0.18 |
| n = 31 | (Significance) | (0.218) | (0.088 | (0.002) | (0.004) | (0.007) | (0.065) |  | (0.011) |  | (0.913) | (0.235) | (0.410) | (0.005) | (0.046) | (0.338) |
|  | |  |  |  |  |  |  |  |  |  |  |  |  |  |  |  |

Note: Spearman correlation coefficients were calculated for the single SNPs applying an additive model where homozygous minor allele carriers were coded as 2, heterozygous coded as 1, and homozygous major allele carriers coded as 0 (= reference category). 11 = homozygous major, 12 = heterozygous, 22 = homozygous minor
